# AUC of Calvert's formula in targeted intra-arterial carboplatin chemoradiotherapy for cancer of the oral cavity

**DOI:** 10.1038/sj.bjc.6601818

**Published:** 2004-04-20

**Authors:** R Oya, S Nakamura, K Ikemura, S Takagi, H Mugino

**Affiliations:** 1 Department of Oral and Maxillofacial Surgery, School of Medicine, University of Occupational and Environmental Health, Kitakyushu, Japan

**Keywords:** AUC, carboplatin, thrombocytopenia, targeted intra-arterial chemotherapy, radiation therapy, oral cavity cancer

## Abstract

We investigated whether intra-arterial administration of carboplatin using Calvert's formula is useful for avoiding thrombocytopenia in targeted chemoradiotherapy in patients with squamous cell cancer of the oral cavity and oropharynx. Carboplatin was infused intra-arterially under digital subtraction angiography in 28 patients. In the first group of patients, the dose of carboplatin was calculated according to the body surface area (BS group). In the second group, the dose was calculated using Calvert's formula (AUC group). The value for AUC (area under concentration *vs* time curve; mg ml^−1^ min^−1^) in the formula was set at 4.5. All patients received concurrent radiotherapy (30 Gy) and were given oral tegafur-uracil (UFT®, 400–600 mg day^−1^). The AUC group showed a significantly lower percentage platelet reduction than the BS group (49.0±22.0 *vs* 65.1±23.2%; *P*=0.045) and also tended to have a higher platelet nadir count (10.9±4.2 *vs* 8.4±5.8 × 10^4^; *P*=0.27) without reducing the antitumour effect. The value of 4.5 for target AUC is recommended clinically. However, AUC of Calvert's formula could not predict thrombocytopenia associated with intra-arterial chemoradiotherapy due to the variability of the actual AUC.

Concurrent intravenous systemic chemotherapy and radiation therapy has been applied to locally advanced squamous cell cancer of the oral cavity and oropharynx (Mohr *et al*, 1994). Several studies have been published using chemoradiotherapy based on intravenous platinum derivatives for advanced head and neck cancer, as a strategy for preoperative treatment or organ preservation (Koch *et al*, 1995; Kirita *et al*, 1996; Wanebo *et al*, 1997).

Recent advances in the angiographic technique have enabled intra-arterial administration of chemotherapeutic agents under fluoroscopic guidance directly through microcatheters to selectively saturate the targeted primary tumour (Robbins *et al*, 1992;Imai *et al*, 1995; Korogi *et al*, 1995). Encouraging results, including high tumour response rates and good local and regional control, have been noted with targeted intra-arterial chemotherapy and concomitant radiation therapy for advanced head and neck cancer (Robbins *et al*, 1997; Oya and Ikemura, 1999).

Carboplatin has been commonly used as one of the chemotherapeutic agents for patients with squamous cell carcinoma of the oral cavity and oropharynx, either in neo-adjuvant chemotherapy or in combination with radiation therapy (Aisner *et al*, 1992; Wanebo *et al*, 1997). Myelosuppression, associated especially with thrombocytopenia, is a major dose-limiting factor in this type of therapy. In order to avoid severe thrombocytopenia, Calvert's formula has been reported as being useful for calculating the intravenously injected dose of carboplatin (Calvert *et al*, 1989). However, it remains uncertain whether a dose for intra-arterial administration determined by Calvert's formula would also be effective in predicting the occurrence of thrombocytopenia. Calvert's formula consists of the area under the blood concentration–time curve (AUC) and patient's renal function. It is unclear as to what value for AUC is appropriate in the formula for intra-arterial administration.

The aim of this study is to investigate the usefulness of Calvert's formula in targeted intra-arterial administration of carboplatin and concurrent radiotherapy, in combination with oral administration of tegafur-uracil (UFT®) (Carmichael *et al*, 2002) to avoid thrombocytopenia and to achieve good treatment response in patients with squamous cell cancer of the oral cavity and oropharynx. Further, we determined an actual value for the AUC of plasma-free platinum by serial sampling from the patients, and the result was compared with the target AUC.

## MATERIALS AND METHODS

A total of 15 patients with histologically proven squamous cell cancer of the oral cavity and oropharynx were treated by targeted intra-arterial administration of carboplatin and concurrent radiotherapy in combination with oral UFT® at our university hospital between April 1995 and April 1997. Carboplatin dose was calculated according to the patient's body surface area (BS group), with the amount of carboplatin (mg) being set at 350 × body surface area (m^2^). In 13 patients treated between May 1997 and August 1998, the dose was calculated using Calvert's formula, with 24-h creatinine clearance (Ccr) being used as a substitute for the glomerular filtration ratio (GFR). As carboplatin is mainly excreted from the kidneys, we considered it appropriate to take into account the patient's renal function when determining the dose, and to expect this change to contribute to the avoidance of thrombocytopenia. In Japan, 24-h Ccr (ml min^−1^) is often substituted for GFR. In order to evaluate the differences between individuals, GFR was compensated using the standard body surface area of Japanese, 1.48 m^2^, and was estimated as 24-h Ccr × patient's body surface area/1.48. Based on the formula, the amount of carboplatin (mg) was set at AUC × (GFR+25) (Calvert *et al*, 1989). The figure for target AUC in the formula was set as 4.5 mg ml^−1^ min^−1^, considering the combined use of radiotherapy and UFT® (AUC group). Characteristics of the two patient groups are summarised in Table 1

**Table 1:** Patient characteristics and degree of thrombocytopaenia in two different carboplatin dose determination groups, for 28 patients with cancer of the oral cavity and oropharynx

**Variables**	**BS group (*n*=15)**	**AUC group (*n*=13)**	***P*-value**
*Gender*			0.29
Male	12	8	
Female	3	5	
			
Age (years)	55–80	43–80	0.42
			
*T stage*			0.01
T2	1	8	
T3	2	0	
T4	12	3	
rT3	0	1	
rT4	0	1	
			
Creatinine clearance (ml min^−1^)	90.2±20.5	90.6±20.0	0.94
Injected dose of carboplatin (mg body^−1^)	542±67	488±107	0.055
			
*Response of the primary tumour*			0.85
CR	9	8	
PR	4	5	
NA	2	0	
			
*WHO thrombocytopaenia grade*			0.7
0	6	7	
1	3	3	
2	2	1	
3	2	2	
4	2	0	

BS=body surface area, AUC=area under concentration–time curve, Plt=platelet, NA=not assessed, T stage is in accordance with the UICC system in 1987.

. TNM stage of the tumour was classified in accordance with the UICC system published in 1987 (Hermanek and Sobin, 1987). During this study, except for the dose-setting methods for carboplatin, we did not change any clinical practice to influence outcomes between the two treatment groups. This study is a retrospective review of clinical practice offered to the patients. Written informed consent was obtained from all patients concerning treatment choice for their tumour.

Intra-arterial carboplatin infusion was carried out through a microcatheter positioned angiographically, targeting only the dominant blood supply of the primary tumour. The injection was performed using an infusion pump, and the rate of infusion was fixed at 150 ml h^−1^. At the end of the carboplatin infusion, intra-arterial methyl prednisolone (125 mg) was administered to prevent intimal damage of the artery containing the microcatheter. Arterial catheterisation was performed by the interventional radiologist, and the catheter was removed following the infusion. Radiation therapy consisted of either conventional external beam X-ray radiation (1.8–2.0 Gy per fraction; two patients) or accelerated hyper fractionation radiation (1.5 Gy per fraction; twice daily; 26 patients). The tumour was radiated with a total dose of 30 Gy. Intra-arterial infusion was administered after an irradiation of about 10 Gy. Tegafur-uracil (UFT®, 400–600 mg day^−1^) was administered orally from the time of performing the diagnostic biopsy to completion of radiotherapy.

Pharmacokinetic sampling for free platinum concentration in the plasma was performed on 12 patients in the AUC group. Blood samples (5 ml) were collected in tubes immediately after carboplatin injection and then at 30 min, 1 h, 2 h, 6 h, and 24 h. Each sample was centrifuged at 3000 rpm for 10 min. Plasma was then placed in an ultrafiltration kit (Centrifree MPS-3; Amicon Inc., Tokyo) and was centrifuged for 15 min at 3000 rpm. This plasma ultrafiltrate was immediately frozen and stored at −20°C. Platinum was determined by flameless atomic absorption spectrophotometry. The AUC of free platinum was calculated using the trapezoidal rule from the concentration–time curve of free platinum in the ultrafiltrate.

The degree of thrombocytopenia was assessed by World Health Organization (WHO) toxicity criteria, platelet nadir count, and the percentage reduction in platelet count. The percentage reduction in platelet count was calculated as [(pretreatment count−nadir count)/pretreatment count] × 100.

After completion of radiotherapy with a single carboplatin infusion, all patients underwent an evaluation to determine the degree of side effects and the clinical response of the tumour including CT and MRI studies. Response criteria were defined as follows: complete response (CR), complete disappearance of the tumour mass; partial response (PR), shrinkage of the tumour mass greater than 50% of the product of the perpendicular diameters; no change (NC), shrinkage of less than partial response.

Differences between the two groups were compared using the Mann–Whitney *U*-test for univariate nonparametric statistical analysis. Spearman correlation coefficients were used to examine the relationship between the percentage reduction, nadir count in platelet and AUC, Ccr, and total dose of administered carboplatin. Data were considered statistically significant if *P*-values were less than 0.05.

## RESULTS

All selected arteries for injection were branches of the external carotid artery, and the mean number of injected arteries was 2.1 vessels per tumour in the BS group, and 1.9 vessels in the AUC group. A total of time of intra-arterial injection to the tumour ranged from 18 to 69 min (mean 35±15 min, median 32 min) in the BS group, and from 13 to 63 min (mean 26±14 min, median 21 min) in the AUC group. The BS group had significantly longer time for injection than the AUC group (*P*=0.04). The total dose of administrated carboplatin was higher in the BS group than in the AUC group. The BS group showed more locally advanced primary tumours than the AUC group (Table 1).

**Figure 1:**
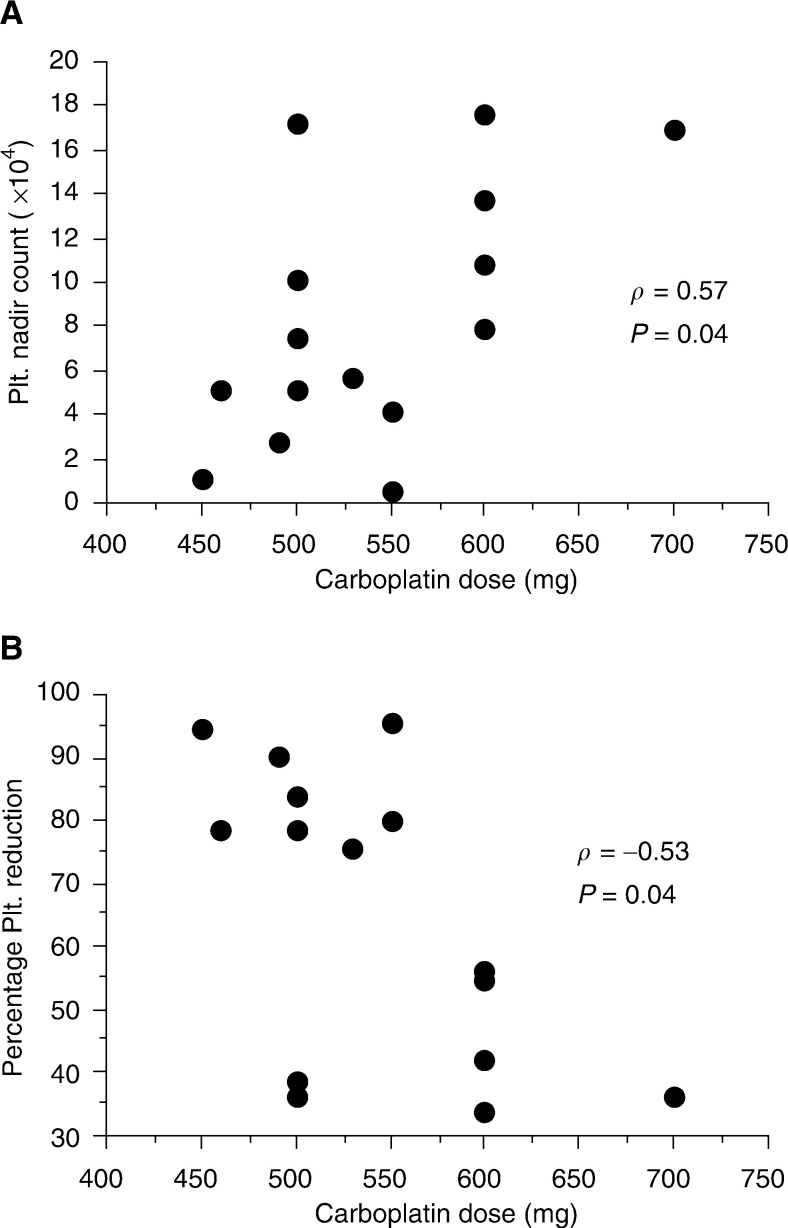
Platelet nadir count (**A**), percentage platelet reduction (**B**) and total dose of carboplatin in the 15 patients injected with 350 mg m^−2^ of carboplatin (BS group).

The relationship between the administered dose of carboplatin and the degree of thrombocytopenia in the BS group is shown in Figure 1

**Figure 2:**
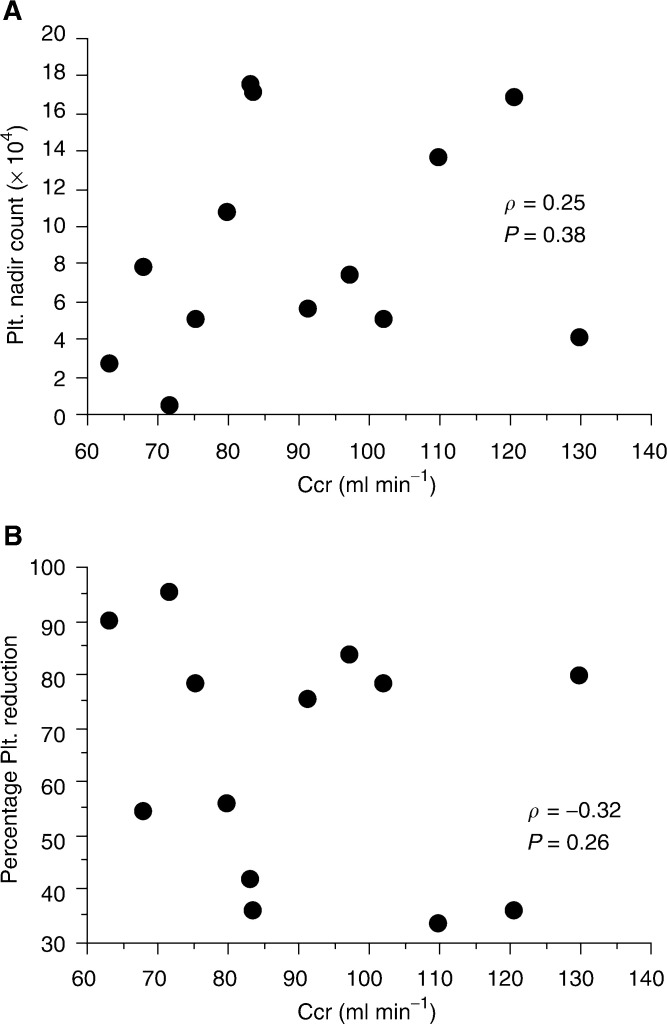
Platelet nadir count (**A**), percentage platelet reduction (**B**) and creatinine clearance in the13 patients injected with 350 mg m^−2^ of carboplatin (BS group).

. A weak linear relationship was observed between the carboplatin dose and the platelet nadir count (*ρ*=0.57; *P*=0.04), and between the dose and the percentage platelet reduction (*ρ*=0.53; *P*=0.04); however, these results do not imply a meaningful correlation, that is, with the increase of the carboplatin dose, the reduction rate decreased and the nadir increased. In 13 patients of the BS group, in whom the pretreatment 24-h Ccr values were known, the figure of Ccr was correlated to the degree of thrombocytopenia. There were no relationships between the Ccr and the platelet nadir count (*ρ*=0.25; *P*=0.38), or between the Ccr and the percentage platelet reduction (*ρ*=-0.32; *P*=0.26), as described in Figure 2

. However, severe thrombocytopenia (the WHO grade 4) was recognised in two of 15 patients (Table 1), and both the two patients revealed a decreased figure of Ccr (63.1, 71.6 ml^−1^ min^−1^, respectively).

Figure 3

**Figure 3:**
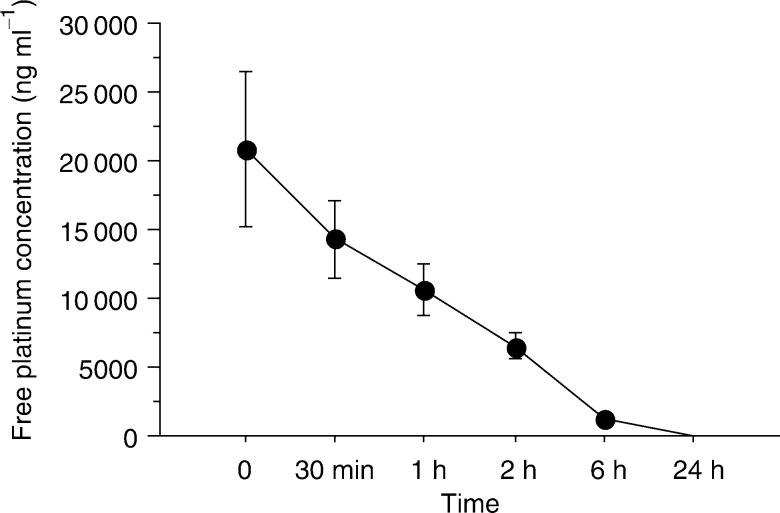
Plasma profile of free platinum in the 12 patients injected with carboplatin with the target AUC set at 4.5 mg ml^−1^ min^−1^ (AUC group).

shows the time curves of plasma ultrafiltrate free platinum concentration (mean±s.d.) in the 12 patients injected with carboplatin, based on a target AUC value of 4.5. The actual AUC of free platinum was found to be 6.5±1.0 mg ml^−1^ min. Figure 4

**Figure 4:**
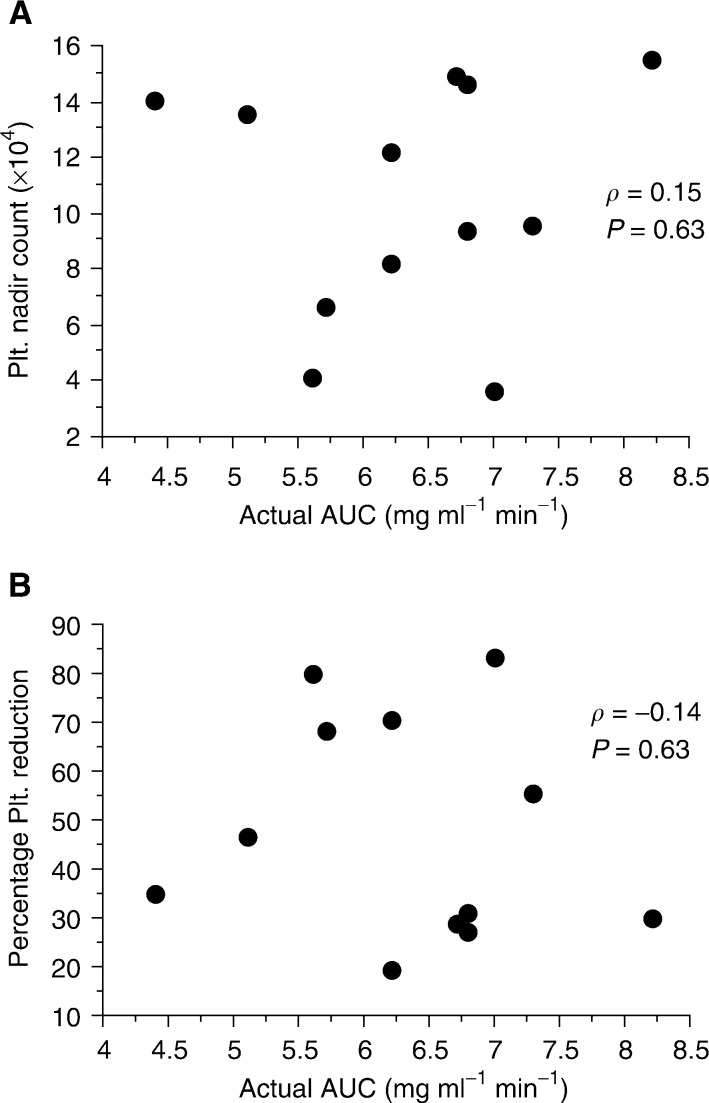
Platelet nadir count (**A**), percentage platelet reduction (**B**), and actual AUC in the 12 patients injected with carboplatin with the target AUC set at 4.5 mg ml^−1^ min^−1^ (AUC group).

shows the relationship between the actual AUC and the degree of thrombocytopenia. There was no correlation between the actual AUC and the platelet nadir count (*ρ*=0.15; *P*=0.63), or between the actual AUC and the percentage platelet reduction (*ρ*=−0.14; *P*=0.63).

**Figure 5:**
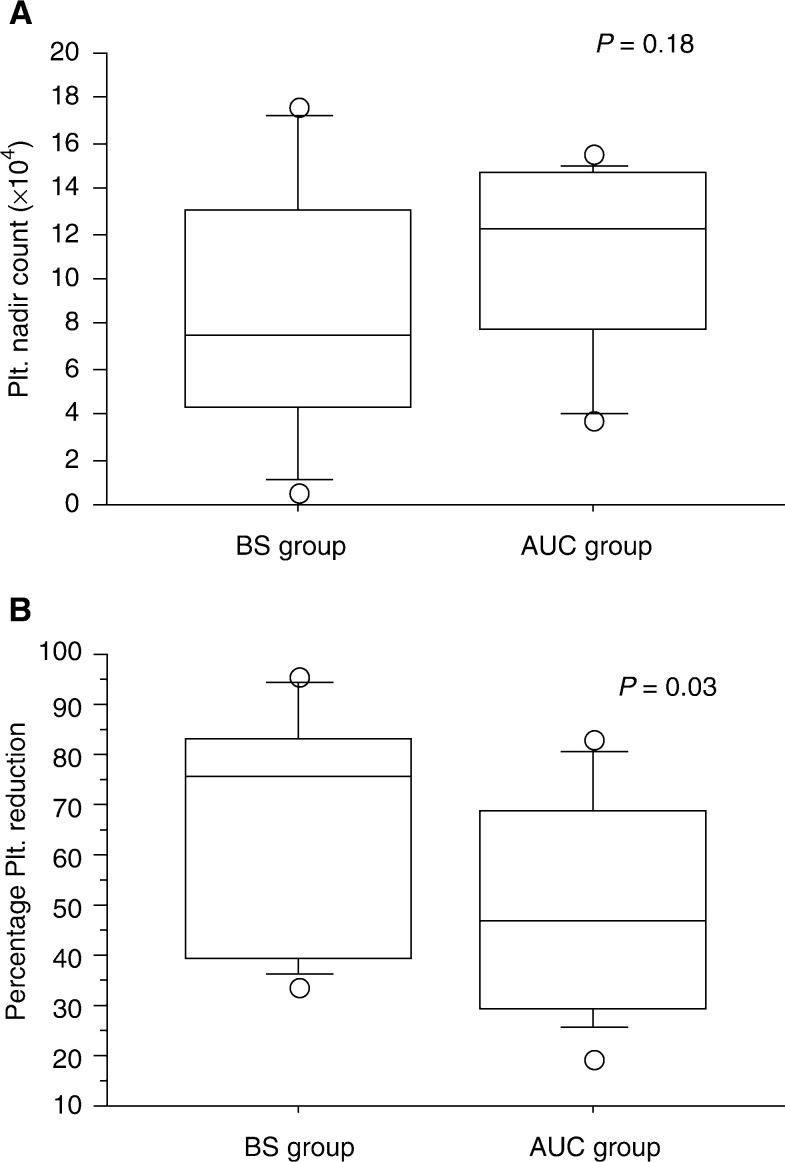
Difference of platelet nadir count (**A**) and percentage platelet reduction (**B**) between the BS and AUC groups.

The percentage reduction in platelet count revealed a significantly lower value in the AUC group than that in the BS group (*P*=0.045). In addition, the AUC group had no occurrence of grade 4 thrombocytopenia, and exhibited a greater nadir count than the BS group (Figure 5

). Otherwise, there were no significant differences between the two groups with respect to the WHO thrombocytopenia grade (*P*=0.70), injected carboplatin dose, and in patient's renal function (24-h Ccr).

A clinical response to the therapy in 26 patients revealed 17 CR (65%) and nine PR (35%). There was no difference in response between the two groups. Two patients in the BS group had not completed planned radiation therapy because they developed severe myelosuppression (leukopenia and thrombocytopenia), and were excluded from the evaluation of clinical response (Table 1).

## DISCUSSION

We have already reported a good clinical and histological result using intra-arterial carboplatin chemoradiotherapy, in which the dose was determined according to the patient's body surface area (Oya and Ikemura, 1999). However, unpredicted severe thrombocytopenia and leukocytopenia developed in three of 15 patients with squamous cell cancer of the oral cavity and oropharynx, and two of these three patients could not complete planned therapy due to secondary complications following myelosuppression (one with sepsis, the other with DIC). Thus, our experience indicated the need for appropriate intra-arterial dose determination of carboplatin.

The major dose-limiting factor for carboplatin therapy has been recognised to be thrombocytopenia. When carboplatin is administrated preoperatively (as in neo-adjuvant or induction chemotherapy), it is essential to predict the development of thrombocytopenia for the successful performance of the planned surgery. In order to avoid severe thrombocytopenia, it has been reported that it is useful to calculate the intravenously injected dose of carboplatin using Calvert's formula, according to both AUC and the renal function of the individual patient. Calvert *et al* (1989) reported that the AUC value is the most important factor in determining the degree of thrombocytopenia and myelosuppression that can be expected in response to a dose of carboplatin. In the administration of carboplatin, either as a single agent or in combination with other drugs, the AUC value has been reported in many cancers to predict the degree of myelosuppression.

UFT also has haematological toxicity (neutropenia, thrombocytopenia). In a previous study in which UFT was used as a single chemotherapy agent for treating colorectal cancer, thrombocytopenia was observed in 18% of cases (33 out of 187) and graded as severe in only one case (Carmichael *et al*, 2002). In the thrombocytopenia observed in our intra-arterial chemoradiotherapy, UFT might have influenced its occurrence and its severity. UFT may also have been directly responsible for the thrombocytopenia; however, we could not evaluate the degree of its contribution to the thrombocytopenia.

Calvert's formula has been shown to be effective for intravenous administration, but its usefulness for intra-arterial injection has remained unknown. We have found no previous reports on the usefulness of AUC for intra-arterial infusion of carboplatin. The degree of thrombocytopenia in the BS group was not associated with the administrated dose of carboplatin (Figure 1). In addition, severe thrombocytopenia (the WHO grade 4) was recognised in two of 15 patients (Table 1), and the two patients showed decreased value of Ccr. These results suggest that the calculated dose of carboplatin according to the patient's body surface area is inappropriate to predict the development of thrombocytopenia, and a dose should be considered with a patient's renal function. The thrombocytopenia in the AUC group showed no association with the actual AUC (Figure 4). These results suggest that the actual AUC value itself in Calvert's formula for the intra-arterial administration of carboplatin could not predict thrombocytopenia due to the variability of the actual AUC. However, the percentage reduction in platelet count revealed a significantly lower value in the AUC group than that in the BS group (*P*=0.045) (Figure 5). This means that an individual dose strategy based on renal function helps to avoid the thrombocytopenia associated with intra-arterial chemoradiotherapy in patients with cancer of the oral cavity and oropharynx.

When determining the appropriate dose of carboplatin, the value set for the target AUC and the precise measurement of GFR are crucial, with respect to both the chemotherapeutic effect and the reduction of myelosuppression. Calvert *et al* proposed that an AUC value of no less than 4 mg ml^−1^ min^−1^, and preferably as high as 5 mg ml^−1^ min^−1^, should be used for intravenous carboplatin when administered in combination with other cytotoxic agents. Setting our target AUC value at 4.5 mg ml^−1^ min^−1^ was therefore considered to be appropriate for the combination of intra-arterial carboplatin, oral UFT, and radiotherapy. This value was supported by the finding that the incidence of thrombocytopenia was reduced without a decrease in the response of the primary tumour, when comparing the BS and AUC groups (Table 1). If the target AUC value is at higher than 4.5 mg ml^−1^ min^−1^, the total injected dose of carboplatin increases and the severity of thrombocytopenia is expected to be worse. We could not predict the difference between the total injected doses of carboplatin between the two groups. From our results for thrombocytopenia and treatment response of the tumours, the value of 4.5 for the target AUC is considered to be appropriate and is recommended for clinical practice. In all, 20 patients with squamous cell carcinoma of the oral cavity and oropharynx have been treated according to the same treatment regimen as the AUC group from September 1998. The results revealed no occurrence of severe thrombocytopenia (WHO grade 4) and good clinical and histological complete response rates (89 and 71%, respectively). From these encouraging results, our targeted intra-arterial chemoradiotherapy could be applied as a strategy for organ preservation or less invasive surgery with acceptable haematological toxicity.

In our pharmacokinetic study, the actual AUC was generally higher than the target AUC. Among the various factors accounting for differences between the actual and target AUC, both carboplatin clearances within the tumour tissue and the time to complete the intra-arterial administration may be important. As a drug is delivered directly to the tumour tissue in target intra-arterial chemotherapy, carboplatin clearance is expected to be influenced largely either by the tumour/normal tissue blood flow ratio, or from the degree of vascularity within the tumour. In order to completely saturate a tumour tissue, multiple arteries must be selected for infusion. This requires time to exchange the microcatheter, and leads to an intermittent administration of carboplatin. In contrast to continuous intravenous infusion, this intermittent administration could account for the difference between the actual and the target AUC value in our intra-arterial chemotherapy.
